# The therapeutic effects of bone marrow-derived mesenchymal stromal cells in the acute lung injury induced by sulfur mustard

**DOI:** 10.1186/s13287-019-1189-x

**Published:** 2019-03-12

**Authors:** Yongwei Feng, Qingqiang Xu, Yuyan Yang, Wenwen Shi, Wenqi Meng, Hao Zhang, Xiaowen He, Mingxue Sun, Yongchun Chen, Jie Zhao, Zhenhong Guo, Kai Xiao

**Affiliations:** 1 Lab of Toxicology and Pharmacology, Faculty of Naval Medicine, Second Military Medical University, Shanghai, 200433 China; 2 National Key Laboratory of Medical Immunology & Institute of Immunology, Second Military Medical University, Shanghai, 200433 China; 3 Origincell Technology Group Co., Ltd., 1118 Halei Rd, Shanghai, 201203 China

**Keywords:** Bone marrow-derived mesenchymal stromal cell, Sulfur mustard, Acute lung injury, Anti-inflammation, Immunomodulation, Tissue repair

## Abstract

**Background:**

Sulfur mustard (SM) is a notorious chemical warfare agent that can cause severe acute lung injury (ALI), in addition to other lesions. Currently, effective medical countermeasures for SM are lacking. Bone marrow-derived mesenchymal stromal cells (BMSCs) possess self-renewal and multipotent differentiation capacity. BMSCs can also migrate to inflammation and injury sites and exert anti-inflammatory and tissue repair functions. Here, we report the curative effect of BMSCs on SM-induced ALI in a mouse model.

**Methods:**

Mice BMSCs were injected into mice via the tail vein 24 h after SM exposure. The distribution of BMSCs in mice was detected by fluorescence imaging. The therapeutic potential of BMSCs was evaluated by the calculating survival rate. The effects of BMSCs on lung tissue injury and repair assessment were examined by staining with H&E and measuring the lung wet/dry weight ratio, BALF protein level, and respiratory function. The effects of BMSCs on the infiltration and phenotypic alteration of inflammatory cells were analyzed by immunohistochemistry and flow cytometry. The levels of chemokines and inflammatory cytokines were examined using the Luminex Performance Assay and ELISA. RNA interference, western blotting, and ELISA were applied to explore the role of the TLR4 signaling pathway in the anti-inflammatory effects of BMSCs. The extent of tissue repair was analyzed by ELISA, western blotting, and immunohistochemistry.

**Results:**

Fluorescence imaging indicated that the lung is the major target organ of BMSCs after injection. The injection of BMSCs significantly improved the survival rate (*p* < 0.05), respiratory function, and related lung damage indexes (wet/dry weight ratio, total proteins in BALF, etc.) in mice. BMSC administration also reduced the level of pro-inflammatory cytokines, chemokines, and inflammatory cell infiltration, as well as affected the balances of M1/M2 and Th17/Treg. Furthermore, solid evidence regarding the effects of BMSCs on the increased secretion of various growth factors, the differentiation of alveolar epithelial cells, and the enhancement of cell barrier functions was also observed.

**Conclusion:**

BMSCs displayed protective effects against SM-induced ALI by alleviating inflammation and promoting tissue repair. The present study provides a strong experimental basis in a mouse model and suggests possible application for future cell therapy.

**Electronic supplementary material:**

The online version of this article (10.1186/s13287-019-1189-x) contains supplementary material, which is available to authorized users.

## Introduction

Sulfur mustard (2,2-dichlorodiethyl sulfide, SM), a highly toxic chemical warfare agent, was first used in World War I in 1917, causing the injury or death of thousands of victims [1]. Due to its availability, stable chemical properties, and ease of production and storage, SM still poses a significant threat to public security today [2]. Exposure to SM can cause damage to the respiratory tract, eyes, skin, and multiple organ systems. Among these organs, pulmonary toxicity appears to be the major cause of mortality and morbidity. Although not fully understood, the primary toxic mechanism of SM is mainly associated with DNA alkylation, inflammatory response, oxidative stress, and proteolytic enzyme activation [3–5]. As the toxic mechanism has not been fully elucidated, there is currently no effective therapeutic treatment for SM exposure.

Bone marrow-derived mesenchymal stromal cells (BMSCs) are multipotent progenitor cells that were first discovered in 1968. BMSCs have self-renewal capacity and the potential to differentiate into bone, fat, cartilage, muscle, tendon, and marrow stroma [6]. Previous studies have shown that, by means of contacting adaptive and innate immune cells, BMSCs could suppress the production of pro-inflammatory cytokines while secreting anti-inflammatory cytokines [7]. In addition to their potential to modulate the immune response and inflammation, BMSCs also have the ability to promote tissue repair following acute lung injury by secreting multiple growth factors essential for function [8].

In recent years, MSCs have attracted massive interest as a potential therapy for acute lung injury. Treatment with intrapulmonary MSCs can significantly decrease endotoxin-induced lung injury and improve survival in mice [9]. Allogeneic hMSC injection can reduce extravascular lung water, improve alveolar capillary permeability, and alleviate pulmonary edema in endotoxin-induced lung injury [10]. Moreover, MSCs were reported to enhance lung repair following ventilation-induced lung injury via a paracrine mechanism [11]. Due to the effective therapeutic effect of BMSCs, we speculate that the administration of BMSCs may provide an effective treatment for the acute lung injury induced by sulfur mustard and become a promising method for clinical application.

In this article, the role of BMSCs in SM-induced lung injury was investigated. First, the effects of BMSCs on the survival rate and pulmonary function were analyzed. Then, we examined whether BMSCs restored SM-induced lung injuries by inhibiting the inflammatory reaction. Furthermore, we also determined whether BMSCs exerted anti-inflammatory effects via inhibiting the TLR4 signaling pathway and further verified these effects in knockout mice. Finally, we explored the tissue repair mechanism of BMSCs.

## Materials and methods

### Preparation of experimental animals

Male ICR mice (25-30 g) were obtained from Sino-British SIPPR/BK Lab. Animal Ltd. (Shanghai, China) and acclimatized for 7 days before their use in our study. Male TLR4 knock-out mice (22–24 g) were obtained from the Model Animal Research Center of Nanjing University. All animal experiments performed in this study conformed to the Guide for the Care and Use of Laboratory Animals and were approved by the Institutional Animal Care and Use Committee.

### SM exposure and treatment

**Fig. 1:**
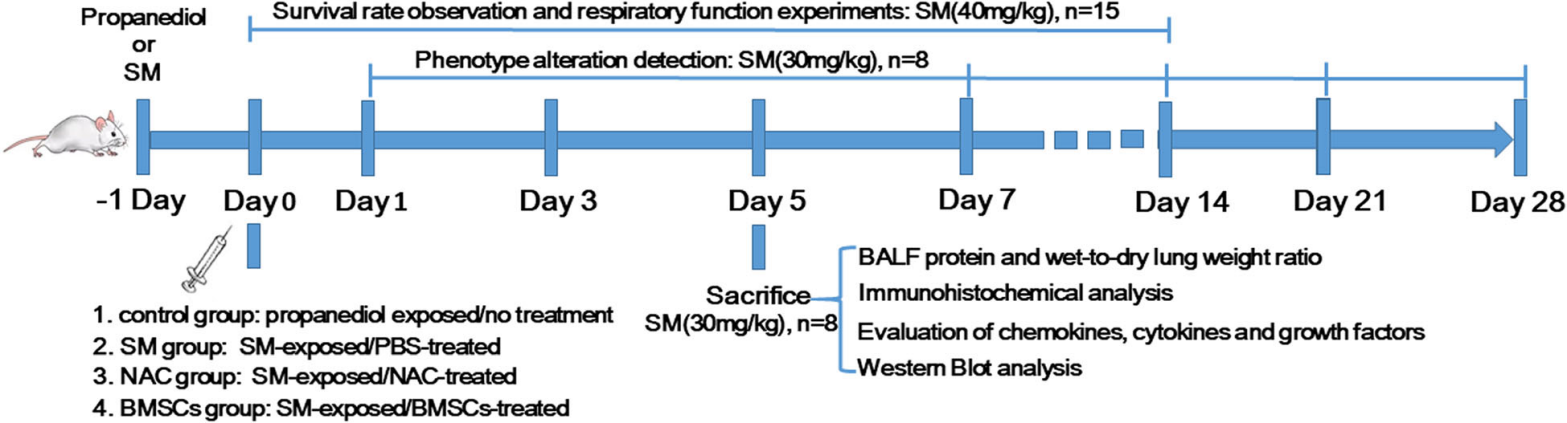
Diagram of the experimental protocol. SM, sulfur mustard; BMSCs, bone marrow-derived mesenchymal stromal cells; NAC, *N*-acetyl cysteine; BALF, bronchoalveolar lavage fluid; PBS, phosphate-buffered saline

SM was formulated into the desired concentration with a propanediol solution (Sigma, St. Louis, MO, USA) before use as described previously [12]. In this research, the mice were randomly divided into four groups: (i) propanediol exposed/no treatment (control group); (ii) SM-exposed/PBS-treated (SM group); (iii) SM-exposed/N-acetyl cysteine (NAC)-treated (SM+NAC group); and (iv) SM-exposed/BMSC-treated (SM+BMSC group). In the control group, the mice were injected with the same volume of propanediol solution. In the SM-exposed group, mice were subcutaneously (SC) injected with SM on the medial and dorsal surface of the skin as reported [13, 14]. Since NAC has been reported to be an efficient antioxidant against SM-induced pulmonary toxicity, it was chosen as a positive control [15]. The mice in the SM+NAC group were given NAC (200 mg/kg) by gavage once per day after SM exposure. The SM+BMSC or PBS-treated group was injected with 1 × 10^7^ BMSCs (resuspended in 200 μl PBS) or the same volume of PBS via the tail vein 1 day after SM exposure. For the survival rate determination and respiratory function experiments, 40 mg/kg SM was applied. For the other experiments in our study, the SM doses were 30 mg/kg. The injection volume was 0.05 ml/10 g in all animals. The mice were either evaluated or sacrificed at the indicated times post-injection, and samples were collected from each mouse for lung injury assessment, immunohistochemical analysis, etc. A diagram of the experimental protocol is shown in Fig. 1.

### Primary culture and identification of BMSCs

BMSCs were obtained from the bone marrow of 3- to 4-week-old male ICR mice and maintained under specific pathogen-free conditions as previously described [16, 17]. Briefly, the bone marrow was flushed from the femurs under aseptic conditions using Dulbecco’s modified Eagle’s medium/F12 (DMEM/F12, ThermoFisher Scientific, USA). The collected cells were washed three times with PBS, resuspended in DMEM/F12 complete medium containing 10% fetal bovine serum (Invitrogen, Carlsbad, CA) and 1% 100 IU/ml penicillin/streptomycin (Gibco, Langley, OK), and seeded at 1 × 10^6^ cells/ml into culture flasks. Cells were cultured in a humidified atmosphere at 37 °C and 5% CO_2_. Nonadherent cells were discarded after 48 h, and the culture medium was changed every 3–4 days thereafter. Cells were harvested when they reached approximately 80–90% confluency and were diluted 1:2 at each passage. The bone marrow cells at the third generation were harvested and characterized.

BMSCs were identified by flow cytometry with a fluorescence-labeled antibody for the positive surface markers CD73, CD90, and CD105 and the negative surface markers CD11b, CD19, CD34, and CD45. BMSCs at the third generation were harvested by trypsin-EDTA. The cells collected after digestion were centrifuged at 1000 rpm and washed with PBS. The BMSC suspensions were incubated with antibodies at room temperature for 30 min in a black chamber. After incubation, flow cytometry (BD FACSCalibur) was conducted and the cells were analyzed using Flow Jo software V10.3 (Flow Jo, LLC, USA). The descriptions of the antibodies used are listed in Additional file 1: Table S1.

The multipotent differentiation potential of BMSCs to differentiate to osteoblasts, adipocytes, and chondroblasts was evaluated. After subculturing to the third generation, and once the cells grew to approximately 70–80% confluency, the culture medium was replaced with osteogenic, adipogenic, or chondrogenic differentiation complete medium (Cyagen Biosciences, China). After the induction of the mineralized osteogenic cultures for 21 days, the accumulation of calcium, intracellular lipids, and mucopolysaccharides were detected by staining with the stains alizarin red S, oil red O, and alcian blue (Sigma-Aldrich, USA), respectively.

### Optical imaging of IR-780 iodide-labeled BMSCs

BMSCs were harvested and resuspended at a concentration of 3 × 10^6^ cells/ml in serum-free medium after reaching 80–90% confluency. Then, the cells were labeled with IR-780 iodide at 20 μM for 20 min and washed three times with PBS. IR-780 iodide-labeled BMSCs were then injected into mice. The mice were sacrificed at the time points of 1 h, 2 h, and 24 h post-injection, and the organs were separated for imaging. A IVIS LaminqII (Caliper LifeSciences, Hopkinton, MA, USA) was used to observe the fluorescence intensity of organs. The exposure time was 1 min.

### Evaluation of the survival rate

The mice in the different treatment groups were observed and carefully compared for 2 weeks. The observed indexes mainly included body weight, dieting, daily activities, hair color, and condition of urination and defecation. These related data were detailed during the survival rate analysis.

### Histopathological examination of lung tissues

After a 5-day period, the animals were anesthetized with intraperitoneal chloral hydrate (4 ml/kg), and their chest was opened by median sternotomy. The lungs were flushed with 10 ml physiological saline and then immediately removed. One of the lungs was fixed in 4% paraformaldehyde for histopathological evaluation and immunohistochemistry. The others were washed with saline and then stored at − 80 °C.

Lungs were first fixed in 4% paraformaldehyde for 24 h. After the processes of dehydration, clearing, wax immersion, and embedding, the lung sections (4 μm) were stained with hematoxylin-eosin (H&E) for histological examination. Histological evaluation was performed by a pathologist blinded to the study groups. The severity of the lung injury was quantified and blindly assessed according to a previously published scoring system by an experienced pathologist based on the images of 10 randomly selected high-power fields [18]. For each section, edema, alveolar and interstitial inflammation, alveolar and interstitial hemorrhage, atelectasis, necrosis, and hyaline membrane formation were each scored using a 0- to 4-point scale according to the scoring system: 0 = no injury; 1 = minimal, low level of PMN infiltration, slight thickening of the alveolar septum, mild bleeding and edema, present in 1–25% of the section; 2 = moderate, present in 25–50% of the section; 3 = severe, present in 50–75% of the section; 4 = maximal, diffuse infiltration of PMN, serious thickening of alveolar septum, severe bleeding and edema, present in 75–100% of the section. A total lung injury score was calculated as the sum of the criteria.

### Measurement of BALF protein and wet-to-dry lung weight ratio

Mice were deeply anesthetized, and their abdomens were opened. After clipping the spinal cord for exsanguination, the trachea was isolated. Then, the thoracic cavity was opened, and the block was removed. The trachea was fixed by forceps and partly snipped by surgical scissors. A tube serving as a catheter for bronchoalveolar lavage was then introduced into the trachea and secured using 2/0 suture. The right lung was lavaged three times with 800 μl phosphate buffer saline, which was collected in centrifuge tubes. The collected BALF was immediately pooled and centrifuged (15 min at 1500 rpm), the supernatant was removed and stored in sterile Eppendorf tubes at − 80 °C. Total protein was then quantified in cell-free BALF using a BCA protein assay kit with bovine serum albumin as the standard. The samples were analyzed at 562 nm using a microplate reader.

In the wet-to-dry lung weight ratio evaluation, the mice were sacrificed, the left lungs were excised, and the wet weight was measured. Then, the lungs were placed in an oven at 72 °C for 72 h to obtain the dry weight. The wet-to-dry lung weight ratio (W/D ratio) was calculated by dividing the wet weight by the dry weight.

### Immunohistochemical analysis

Lung tissue sections were first deparaffinized and dehydrated, and then sections were subjected to antigen retrieval by microwave treatment in boiling 0.01 M citrate buffer (pH 6.0) for 20 min. The endogenous peroxidase activity was blocked by 3% hydrogen peroxide for 10 min. Sections were incubated with goat serum (room temperature, 20 min) to block nonspecific binding followed by an overnight incubation at 4 °C with 50 μl anti-myeloperoxidase antibody (the neutrophil marker), anti-CD3 antibody (the T lymphocyte marker), anti-CD68 antibody (the macrophage marker), or anti-Ki-67 antibody (the proliferating cell marker). Sections were then incubated with a secondary antibody for 1 h and horseradish peroxidase (HRP) streptavidin for 30 min at 37 °C with DAB as the substrate. The sections were examined with an Olympus IX71 microscope (Olympus Co., Tokyo, Japan). The proportion of positive cells from 10 randomly selected microscopic fields was quantified. For Ki-67 proliferative quantification, the microscopic fields were selected from the same tissue site within each specimen in a blinded manner. The proliferative index was calculated as the number of positively stained nuclei on each slide. The presence of any nuclear staining, regardless of intensity, was considered positive. The descriptions of the antibodies used are listed in Additional file 1: Table S1.

### Flow cytometry

BALF was harvested as described above, and approximately 10^6^ lung cells were first suspended in 1 ml washing buffer (0.5% BSA in PBS) and then subjected to a surface antibody staining solution containing a mix of anti-mouse F4/80, anti-mouse CD86, or anti-mouse CD206 (MMR). Splenic lymphocytes were extracted by using a mouse spleen lymphocyte separation reagent kit (LTS1092PK, TBD sciences) after labeling the surface antibody anti-mouse CD4. The splenic lymphocytes were then fixed and permeabilized using a True-Nuclear™ Transcription Factor Buffer Set kit (Biolegend, Cat# 424401) prior to performing intracellular staining with anti-mouse FoxP3 or anti-mouse RORγT. After incubation, cells were washed twice and finally suspended in FACS buffer for flow cytometry analysis. The data were collected using BD Biosciences software. Data were analyzed using FlowJo Software V10.3.

### Evaluation of chemokines with Luminex Performance Assays

Serum samples were collected by centrifuging blood at 12000 rpm for 10 min, and the samples were stored at − 80 °C until use. The expression of chemokines, including CCL2, CCL3, CCL4, CCL5, CXCL1, CXCL2, CXCL5, CXCL9, CXCL10, and M-CSF, was measured by a MILLIPLEX MAP Mouse Cytokine/Chemokine Magnetic Bead Panel 96-Well Plate Assay using a Luminex Flexmap 3D Multiplexing Instrument according to the manufacturer’s instructions. Briefly, the beads (included in the kit) were incubated with buffer, chemokine standards or samples in a 96-well plate at 4 °C overnight, and further incubations with the detection antibody and streptavidin-phycoerythrin were conducted at room temperature on a plate shaker. The plate was analyzed using Luminex FLEXMAP 3D with xPONENT software. Finally, the median fluorescent intensity (MFI) data were saved and analyzed using a 5-parameter logistic or spline curve-fitting method for calculating chemokine concentrations in samples.

### Evaluation of cytokines and growth factors with ELISA

The levels of TNF-α, IL-1β, IL-6, IL-10, IL-12, IL-17, TGF-β, epidermal growth factor (EGF), fibroblast growth factor (FGF), and platelet-derived growth factor (PDGF) were measured using an enzyme-linked immunosorbent assay (ELISA) kit employing the quantitative sandwich enzyme immunoassay technique. Taking TNF-α as an example (the other molecules were measured by the same method), the tissue was homogenized and centrifuged at 3500 rpm for 10 min. Then, the supernatant was obtained and added to TNF-α antibody-coated micro wells (included with the kit) together with the biotin-conjugated secondary antibody and incubated for 90 min at 37 °C. After washing five times, the wells were incubated with HRP-streptavidin complex for 30 min at 37 °C. Then, the washing process was repeated, and color reactions were conducted during an incubation for 30 min at 37 °C in the dark. Finally, the optical density was measured by using an ELISA plate reader after the addition of stop solution. The absorbance was read at 450 nm.

In the cell model, RAW264.7 cells (a macrophage cell line) and splenic lymphocytes were precultured in 12-well plates at a confluent density. SM was diluted in DMEM at a concentration of 50 μM and then immediately added to the cell media for 30 min. BMSCs alone and the culture supernatant of BMSCs were added after SM exposure. At different time periods (24, 48, 72, 168 h), the concentrations of cytokines in the supernatant of cocultures were measured by ELISA.

### Western blot analysis

Frozen lung samples or cells were harvested and lysed in RIPA buffer containing protease inhibitors. The protein concentration was determined with the BCA assay kit. The tissue homogenate and cells were then centrifuged at 12000*g* at 4 °C for 5 min, and the supernatants were collected to detect the target protein. Protein extracts were loaded into the lanes of sodium dodecyl sulfate-polyacrylamide gel electrophoresis (SDS-PAGE) gels, and the polypeptides were separated by electrophoresis and transferred onto polyvinylidene fluoride (PVDF) membranes. The PVDF membranes were blocked in 5% skim milk for 60 min and incubated overnight at 4 °C with antibodies against TLR4, NFκB p65, AQP-5, SP-C, VE-Cadherin, Occludin, Claudin-5, ZO-1, and GAPDH. The descriptions of the antibodies used are listed in Additional file 1: Table S1. Then, the membranes were incubated with a secondary antibody at room temperature for 30 min after being washed in TBST three times. The chemiluminescence reaction was conducted with an ECL kit for 1 min, and the protein bands were scanned and quantified based on optical densities using ImageJ (version 1. 34 s) and normalized to GAPDH. The values shown are the means of three independent experiments.

### Statistical analysis

All data are presented as the mean ± standard deviation. Statistical significance was determined by Student’s 푡 test or ANOVA, and *p* < 0.05 values were considered statistically significant. Statistical analysis was performed using SPSS21.0 software.

## Results

### Characterization of BMSCs

**Fig. 2:**
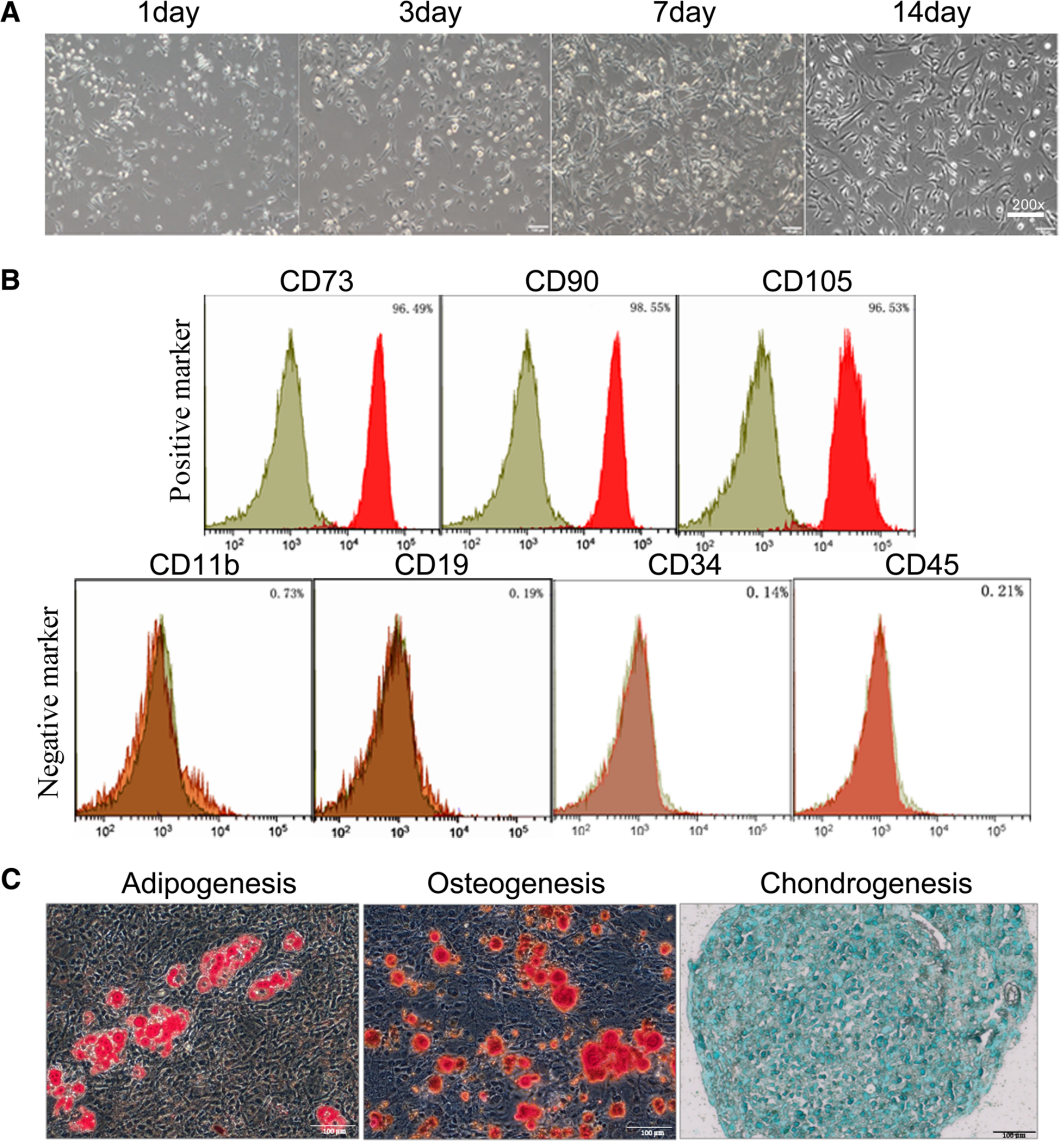
Isolation and characterization of mouse bone marrow-derived mesenchymal stromal cells. **a** Morphology of BMSCs at different time points (1 day, 3 days, 7 days, 14 days) after seeding (× 200). **b** Immune-phenotype of BMSCs positive for CD73, CD90, and CD105 and negative for CD11b, CD19, CD34, and CD45. **c** Oil red O staining of adipo-induced BMSCs, alizarin red staining of osteo-induced BMSCs and toluidine blue staining of chondro-induced BMSCs

BMSCs were cultured as described in the “Materials and methods” section. BMSCs were identified based on typical morphological observations with an inverted microscope, phenotypic determination with flow cytometry and multipotent differentiation capacity. Throughout the 2-week cultivation, the cells were spindle shaped and exhibited a swirling distribution (Fig. 2a). The BMSC markers were determined by flow cytometry analyses (Fig. 2b). The results showed that the cells stained positive for CD73 (96.49%), CD90 (98.55%), and CD105 (96.53%) and stained negative for CD11b (0.73%), CD19 (0.19%), CD34 (0.09%) and CD45 (0.01%). The adipogenesis, osteogenesis, and chondrogenesis potential of the cells were confirmed by red oil O staining, alizarin red staining, and toluidine blue staining, respectively (Fig. 2c).

### BMSCs rescued SM-induced lung injury

**Fig. 3:**
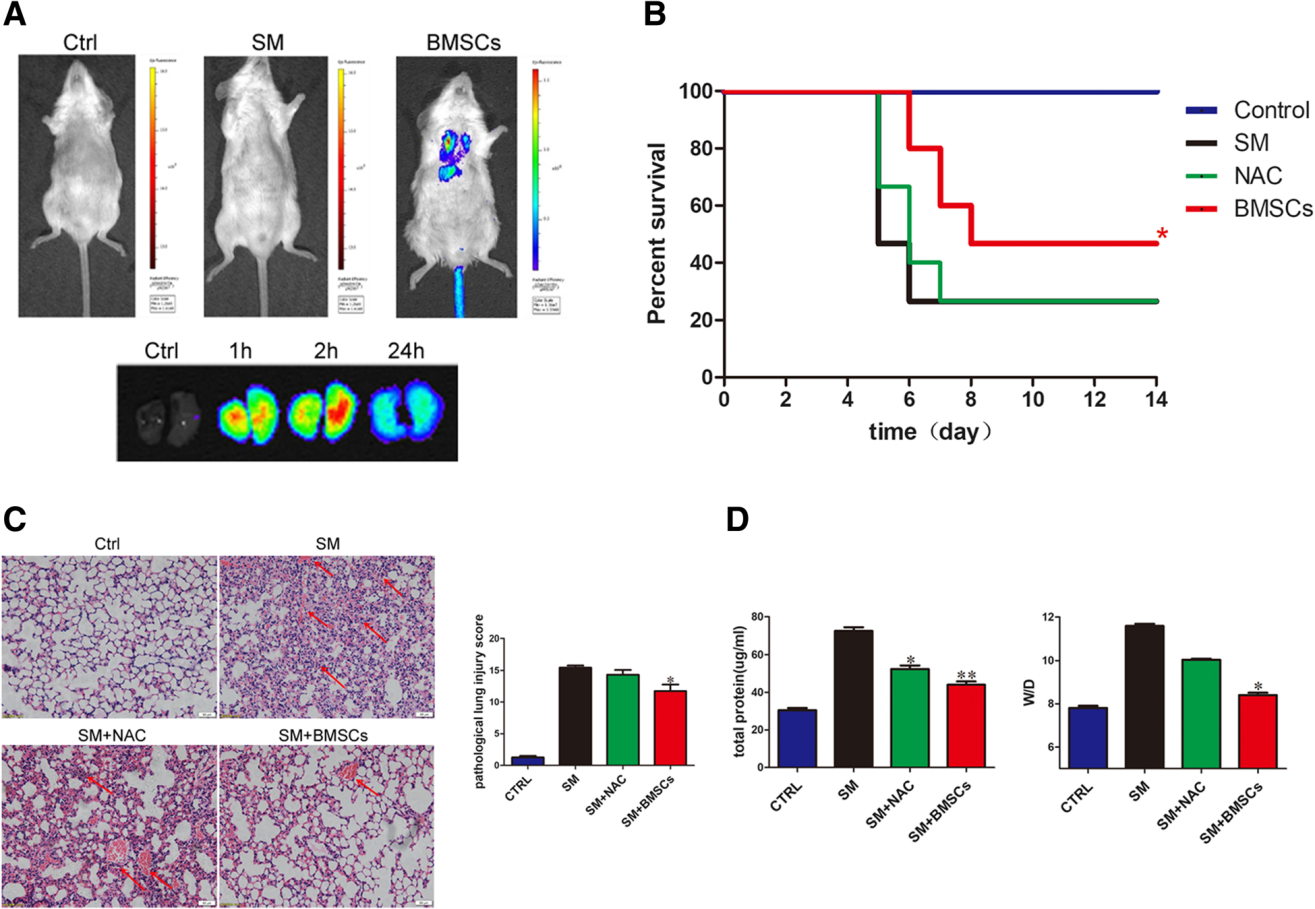
BMSCs rescued SM-induced lung failure. **a** In vivo and in vitro imaging of IR-780 iodide-labeled BMSCs after transplantation. Imaging 1 h after BMSC injection in SM-poisoned mice and at different time points after BMSC injection in SM-poisoned mouse lungs. **b** Evaluation of the influence of BMSCs on the survival rate of SM-treated mice. *p < 0.05 vs SM group (*n* = 15). **c** Representative photographs of lung sections stained with H&E from SM-exposed mice (× 200). A normal mouse lung tissue included the following: normal alveolar cavities, no edema, and hemorrhage. A representative image of the SM group included the following: a high level of inflammatory cell infiltration present in the alveolar cavity, mucosal epithelium, and airways; edema and hemorrhage in many areas; and marked thickening of the alveolar septum. Little improvement was observed in the NAC group; the BMSC group showed conspicuous protection against SM-induced damage to the lung tissue, as revealed by the relatively normal alveolar cavity, mucosal epithelium and airways as well as minimal inflammatory cell infiltration and septal thickening (left channel). Comparison of pathological lung injury scores in SM-exposed mice. *p < 0.05 vs SM group (right channel). **d** Comparison of BALF protein level (left channel) and wet-to-dry lung weight ratio (right channel) in SM-exposed mice. *p < 0.05 compared with the SM group; **p< 0.01 compared with the SM group

To detect the distribution of BMSCs in SM-injured mice, fluorescent imaging in vivo was conducted. As shown in Fig. 3a, the fluorescence intensity in SM-treated lungs showed a slight increase 1 h after injection and reached its peak 2 h after injection; most importantly, fluorescent staining could be clearly detected 24 h after injection. Additionally, the fluorescence intensity was higher in the lung than in other parts of the body. These results enable us to acknowledge that the lung was the main target organ after BMSC injection in the SM-poisoned mice.

To assess the therapeutic potential of BMSCs, the daily life behaviors of the mice were recorded. Twenty-four hours after SM injection, decreased eating and physical activity were clearly observed. On the third day, the mice suffered significant weight and appetite loss. A gradual increase in mortality of the mice was observed up to the fifth day. After day 5, the body weight increased, and no mice died after the eighth day. Additionally, the administration of BMSCs alleviated these SM-induced effects. The fundamental state and survival rate were markedly improved, indicating that BMSC injection could significantly improve the survival rate (*p* < 0.05; Fig. 3b).

H&E staining of lung sections was also performed for histological evaluation of the mice. Compared with the SM group and the NAC group, the BMSC-treated mice had significantly decreased tissue lesions and perivascular infiltrates, and the observation of mild bleeding and edema revealed impressive protective effect (Fig. 3c left channel). In addition, the tissue sections were assessed by a histological score. A significantly higher histological score was observed in the SM group. After treatment with BMSCs, the score was predominantly decreased. Additionally, the NAC-treated group did not show a significant difference from control (Fig. 3c right channel). These results demonstrated that BMSCs played an important role in the amelioration of SM-induced lung injuries.

The total protein in alveolar lavage fluid and the wet-to-dry weight ratio are important indicators for measuring the exudation of pulmonary edema. Mice treated with BMSCs showed a decreasing trend in BALF protein levels compared with the mice in the SM group (42.42 ± 1.94 versus 72.49 ± 4.83, *p* < 0.01) (Fig. 3d left channel). Moreover, a decreased W/D ratio was found in the mice receiving BMSC treatment (8.40 ± 0.27 versus 11.89 ± 0.25, *p* < 0.05) compared with the mice in the SM group (Fig. 3d right channel). In addition, BMSC treatment improved lung function, further confirming the therapeutic effect of BMSC administration in mice with SM poisoning (Additional file 2: Figure S1).

### BMSCs regulate the phenotypic alteration of inflammatory cells

**Fig. 4:**
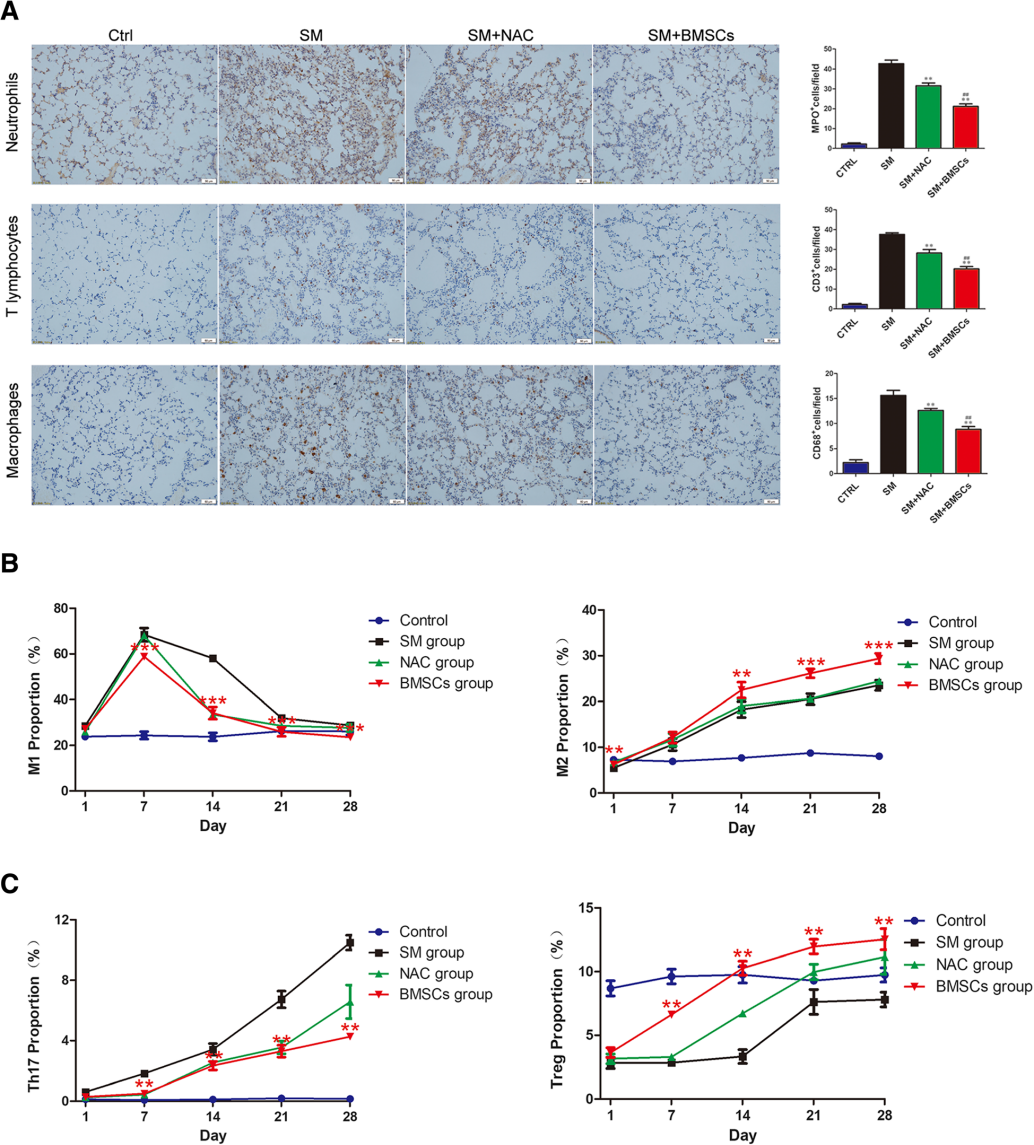
BMSCs regulated the phenotypic alteration of inflammatory cells. **a** Number of MPO-, CD3-, and CD68-positive cells per field of view. **b** BMSCs decrease the polarization of M1 macrophages. ***p < 0.001 compared with the SM group. BMSCs promote the polarization of M2 macrophages. ***p< 0.001 compared with the SM group. **p< 0.05 compared with the SM group. **c** BMSCs decrease the polarization of Th17 cells. **p< 0.01 compared with the SM group. BMSCs promote the polarization of Treg cells. **p < 0.01 compared with the SM group

The infiltration of neutrophils, T lymphocytes and macrophages was assessed. Immunohistochemistry assays revealed that SM injection resulted in the marked upregulation of neutrophils, T lymphocytes, and macrophages, while the administration of BMSCs led to a dramatic decrease in the presence of these cells. In addition, relatively low expression levels were detected in the NAC group (Fig. 4a).

To further investigate the potential interaction between BMSCs and macrophages, the subpopulation change in the BALF and alveolar macrophages were harvested and evaluated by flow cytometry. The proportion of pro-inflammatory M1 macrophages (F4/80^+^ CD86^+^) in the SM group was markedly increased, particularly on day 7. Compared with the SM group, BMSC treatment significantly decreased the percentage of M1 macrophages. At the same time, the percentage of anti-inflammatory M2 macrophages (F4/80^+^ CD206^+^) in the BMSC group was significantly increased compared with the SM group (Fig. 4b, Additional file 3: Figure S2). Thus, BMSCs can exert their anti-inflammatory effects by inhibiting the differentiation of macrophages into pro-inflammatory M1 macrophages and promoting the differentiation of macrophages to anti-inflammatory M2 macrophages.

To assess the effect of SM exposure on Th17/Treg cell expansion, spleen lymphocytes were collected for flow cytometry analysis. The proportion of Th17 cells (CD4^+^ RORγT^+^) in spleen lymphocytes was higher in the SM group than in the control group. Additionally, the proportion of Tregs (CD4^+^Foxp3^+^) in spleen lymphocytes was markedly lower in the SM group than in the control group. The injection of BMSCs slightly decreased the proportion of Th17 cells while increasing the proportion of Treg cells (Fig. 4c, Additional file 4: Figure S3). These results showed that BMSCs can exert their anti-inflammatory effects by inhibiting the differentiation of lymphocytes into pro-inflammatory Th17 cells and promoting the differentiation of macrophages into anti-inflammatory Treg cells.

### BMSCs alleviated the SM-induced inflammatory response in the lung

**Fig. 5:**
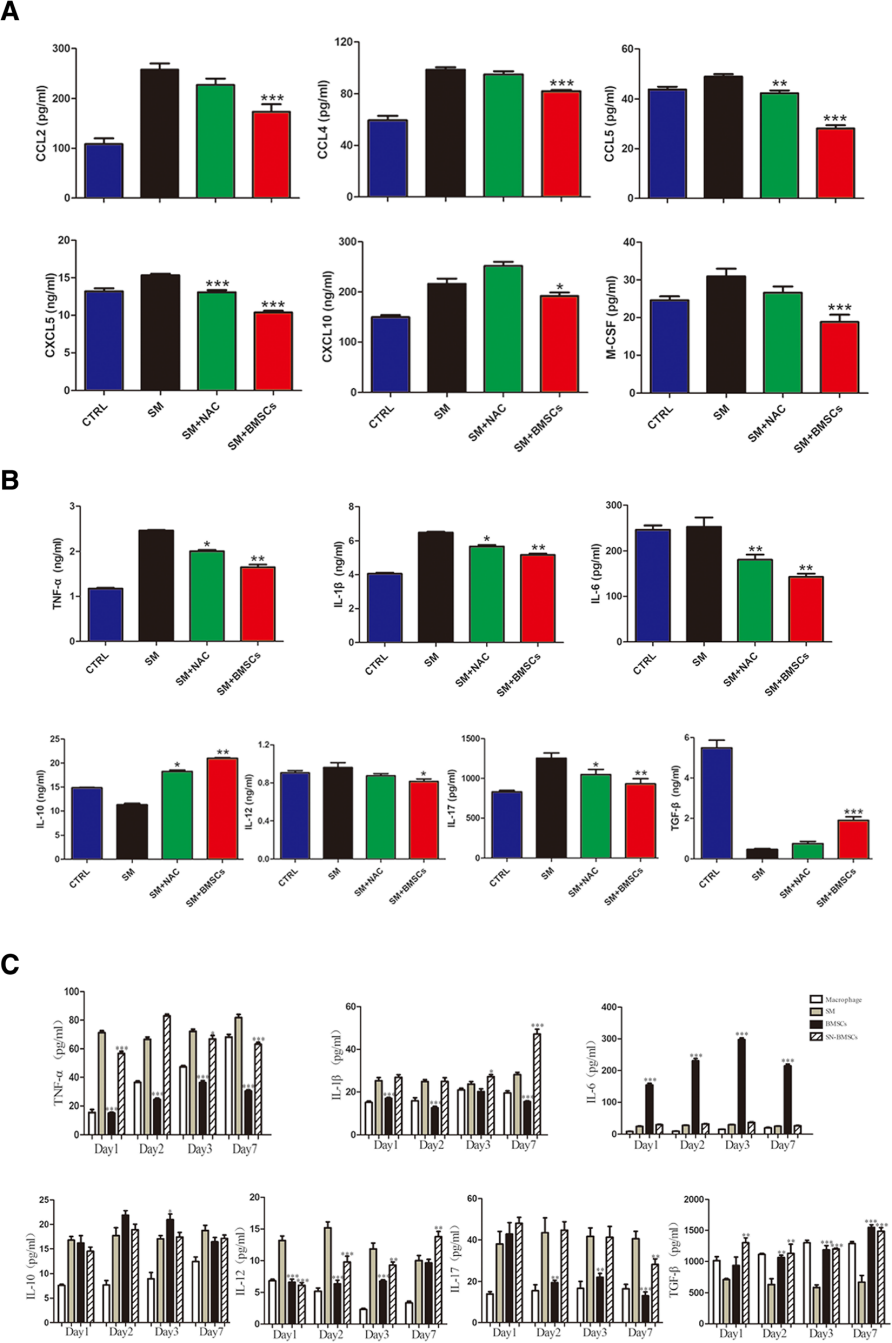
BMSCs alleviated the SM-induced inflammatory response in the lung. **a** Comparison of CCL2, CCL4, CCL5, CXCL5, CXCL10, and M-CSF in SM-exposed mice by Luminex Performance Assays. *p < 0.05 compared with the SM group. **b** Comparison of TNF-α, IL-1β, IL-6, IL-10, IL-12, IL-17, and TGF-β in the lung tissue of SM-exposed mice as determined by ELISA. **c** Expression of the cytokines TNF-α, IL-1β, IL-6, IL-10, IL-12, IL-17, and TGF-β in BMSCs cocultured with Raw264.7 cells or splenic cells after SM exposure. *p < 0.05 compared with the SM group; **p < 0.01 compared with the SM group; ***p < 0.001 compared with the SM group

To investigate the SM-induced inflammatory response in the lung, we screened the expression of chemokines in serum samples with a Luminex Performance Assay. SM induced significant increases in the expression of pro-inflammatory chemokines in the serum. The BMSC injection could markedly reduce the expression levels of CXCL5, CXCL10, CCL2, CCL4, CCL5, and M-CSF (Fig. 5a), while it had no apparent influence on the expression levels of CXCL1, CXCL2, CXCL9, and CCL3.

The expression levels of pro-inflammatory and anti-inflammatory cytokines in the lung tissues were further examined by ELISA. As depicted in Fig. 5b, mice treated with SM exhibited upregulated TNF-α, IL-1β, IL-6, IL-12, and IL-17 expression and downregulated IL-10 and TGF-β expression. However, the expression levels of TNF-α, IL-1β, IL-6, IL-12, and IL-17 in the BMSC group were decreased while expression levels of the anti-inflammatory cytokines IL-10 and TGF-β were significantly increased (Fig. 5b).

Since BMSCs can induce significant changes in cytokines in lung tissue, we continued to test the effects of BMSCs and BMSC supernatants (SN-BMSCs) on cytokine production by coculture with Raw264.7 cells or splenic cells. As shown in Fig. 4c, SM stimulation produced a significant increase in TNF-α, IL-1, IL-6, IL-10, IL-12, and IL-17 and a decrease in TGF-β. Coculture with BMSCs could inhibit the production of TNF-α, IL-1, IL-12, and IL-17, while the levels of IL-6, IL-10, and TGF-β were increased. Additionally, there was no significant difference between the BMSC supernatant group and SM group (Fig. 5c).

### BMSCs suppressed SM-induced inflammation by the targeting TLR4 signaling pathway

**Fig. 6:**
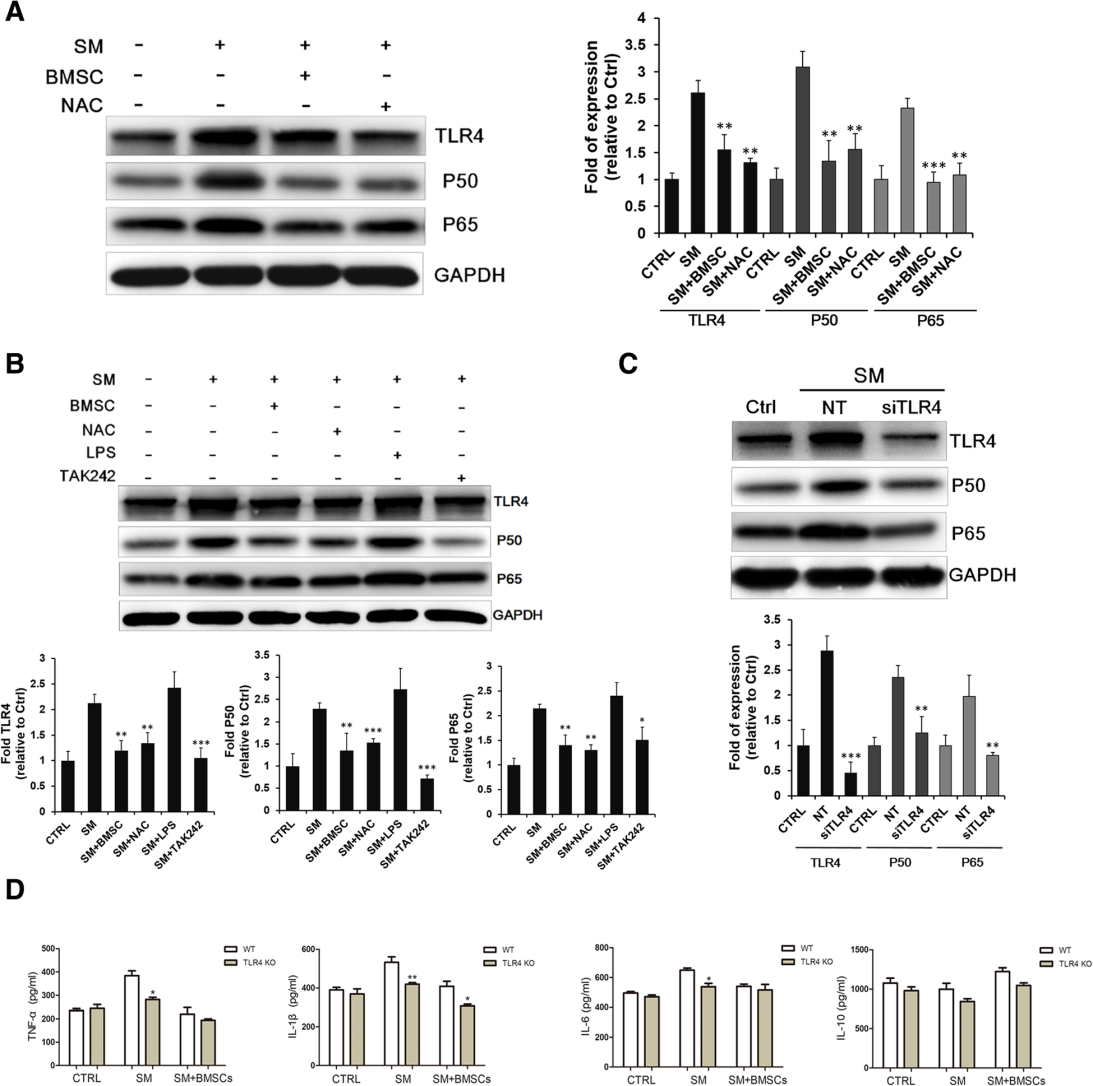
BMSCs suppressed SM-induced inflammation by targeting the TLR4 signaling pathway. TRL4, NFκB p50 and NFκB p65 expression in lung tissue (**a**) and in the Raw264.7 cell model (**b**, **c**). **d** Comparison of TNF-α, IL-1β, IL-6, and IL-10 in the lung tissue of SM-exposed wild type and TLR4 knockout mice as determined by ELISA. *p < 0.05 compared with the WT group; **p < 0.01 compared with the WT group

As described earlier, macrophages may play an important role in BMSC-induced anti-inflammatory effects via the secretion of cytokines; thus, we next explored the potential mechanism of this effect. At the animal level, SM stimulation upregulated the expression of TLR4, NF-*κ*B p50, and NF-*κ*B p65 in lung tissues. BMSC treatment successfully reduced TLR4, NF-*κ*B p50 and NF-*κ*B p65 expression compared with the SM group (Fig. 6a). In the Raw264.7 cell model, the administration of BMSCs had a similar effect; the levels of TLR4 and its downstream targets, which were increased by SM administration, were downregulated with BMSC treatment. TAK-242, an inhibitor of TLR4, could also lower the expression of TLR4 signaling in murine macrophages (Fig. 6b). Moreover, in the Raw264.7 cells transfected with small interfering RNA (siTLR4), the SM-induced upregulation of TLR4 was almost completely abolished (Fig. 6c). These results showed that TLR4 played an important role in the activation of the inflammatory response induced by SM and that BMSCs could suppress inflammation by inhibiting the TLR4 and NF-*κ*B signaling pathways.

To further confirm the role of the TLR4 pathway in SM-induced lung injury, we examined the expression of inflammatory cytokines in a TLR4 KO mouse model. As shown in Fig. 6d, the expression levels of TNF-α, IL-1β, and IL-6 in SM-poisoned wild-type C57 mice were markedly increased compared to those in the normal group, and this effect was blocked in the TLR4 knockout mice. After the administration of BMSCs, the expression of these inflammatory cytokines was further reduced in both wild type and TLR4 knockout mice. Moreover, compared with the SM group, IL-10 was still increased after BMSC treatment, but there was no significant difference in IL-10 levels between the WT and TLR4 knockout mice (Fig. 6d).

### BMSCs promoted the repair of SM-induced lung injury

**Fig. 7:**
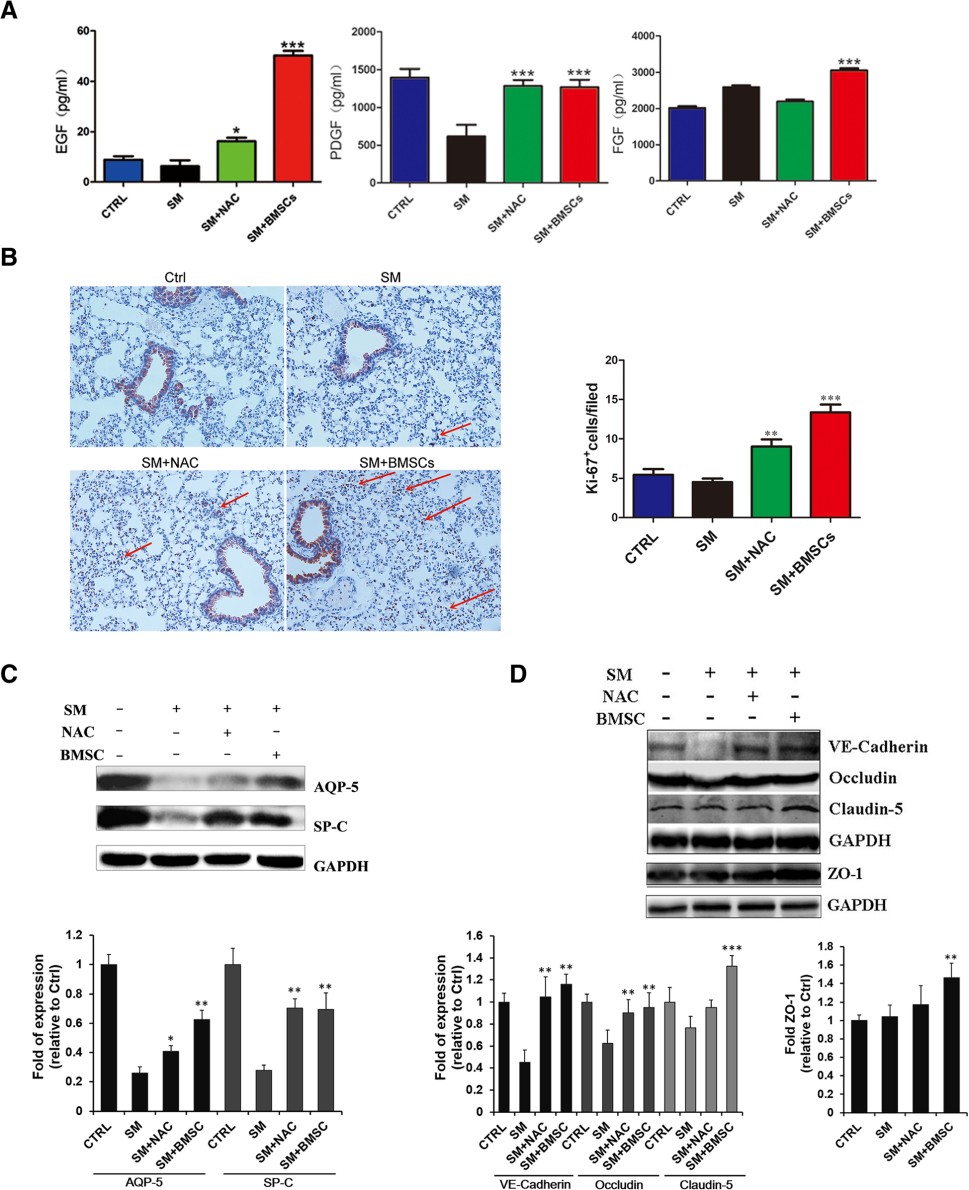
BMSCs promoted the repair of SM-induced lung injury. **a** Comparison of EGF, PDGF, and FGF in the lung tissue of SM-exposed mice as determined by ELISA. *p < 0.05 compared with the SM group; ***p < 0.001 compared with the SM group. **b** Lung section with Ki-67 staining after SM injection (× 200). The red arrow points to the cell that stained positive for Ki-67. **c** AQP-5 and SP-C expression in lung tissue. **d** Cell junction-related protein expression in lung tissue

A previous study reported the BMSC function in tissue repair and regeneration by secreting different kinds of growth factors. Here, the concentrations of epidermal growth factor (EGF), fibroblast growth factor (FGF), and platelet-derived growth factor (PDGF) were measured in lung tissues. The expression levels of EGF and PDGF in the SM group were lower than those of the control group, while the FGF expression in the SM group was higher than that of the control group. Consistent with previous studies, the levels of EGF, FGF, and PDGF in lung tissue were all increased after BMSC treatment (Fig. 7a). Ki-67 staining was conducted to further investigate the differentiation effect of BMSCs. As shown in Fig. 7b, the proliferative activity of both fibroblasts and epithelial cells was markedly lower in the SM group than in controls. The highest level of proliferative activity was found among all the groups after BMSC injection. Besides, we detected the expression of AQP-5 and SP-C (the cell markers of AEC-I and AEC-II) to clarify the mechanisms underlying the function of BMSCs in epithelial repair. The results showed that the AQP-5 and SP-C proteins were markedly upregulated in the BMSC group, indicating that BMSCs promote the re-epithelization and recovery of alveolar epithelial cells (Fig. 7c). In addition, the levels of the cell junction proteins VE-Cadherin, Occludin, Claudin-5, and ZO-1 were quantified. The expression levels of VE-Cadherin and Occludin were significantly decreased in the SM group; Claudin-5 and ZO-1 were not significantly changed. However, the expression levels of VE-Cadherin, Occludin, Claudin-5, and ZO-1 were significantly increased after treatment with BMSCs, showing that BMSCs might reduce pulmonary edema by strengthening the adhesion and tight junction between cells (Fig. 7d).

## Discussion

As a kind of vesicant and alkylating chemical warfare agent, sulfur mustard remains a significant threat for public security due to the ease of its synthesis and handling. As the mechanism of toxicity has not been completely understood, no effective treatment or antidotes are available to eliminate the toxic effect of SM. BMSCs have emerged as a promising therapy for ALI because of various functions, such as their abilities to migrate the injury site, regulate alveolar epithelial permeability, modulate immune responses, and repair tissues [19]. Previous studies have shown the protective and reparative effects of BMSCs in models of lung injury caused by LPS, bleomycin, radiation, etc. [20–22]. Recently, MSCs were shown to be highly resistant to SM exposure and have therapeutic effects in SM-induced injury; however, the exact mechanism by which MSCs play these pivotal roles, especially in SM-induced acute lung injury (ALI), remains to be elucidated [23, 24]. In the current study, we proved that BMSCs could serve as a potential treatment for SM-induced lung injury. Our study assessed the anti-inflammatory and tissue repair effects of BMSCs on SM-induced injuries in mouse lungs and immune cells and further explored the possible mechanism involved in these effects.

SM inhalation could result in extensive pathological changes in the respiratory tract, including the trachea, bronchi, and lung lobes [25–27]. The infiltration of protein-rich edema fluid into the perivascular space, as a result of increased permeability of the alveolar-epithelial barrier, was the feature of acute ALI [28]. In our SM-induced lung injury model, these pathological changes, together with the elevated expression of total protein and W/D weight ratio, were confirmed. In the SM-poisoned mice that received BMSCs, decreased levels of pulmonary edema and inflammatory cells infiltration into alveoli were observed. We also found decreased BALF protein level and W/D lung weight ratio. Recently, BMSCs were reported to be capable of forming connexin 43-containing gap junctional channels and releasing mitochondria-containing microvesicles, which were engulfed by epithelium cells and led to a reduction in plasma exudation [29]. Furthermore, keratinocyte growth factor, the seventh member of the fibroblast growth factor family, was also a crucial paracrine soluble factor that mediated the repair of epithelium cells by BMSCs [10, 11]. Therefore, we hypothesized that the therapeutic effect of BMSCs on SM inhalation is partly due to a reduction in lung vascular permeability. In addition, according to the histological analysis, BMSCs could markedly reduce the infiltration of inflammatory cells, including neutrophils, T lymphocytes, and macrophages, into lung tissues. Inflammatory responses were believed to have a pivotal role in the pathology of SM poisoning [30–32]. We hypothesized that the benefit of BMSC treatment was also mediated through the suppression of inflammatory responses.

Chemokines play a key role in regulating the trafficking of immune cells during inflammatory responses. Previous studies showed elevated levels of CCL2, CCL3, and CCL4 in patients with mustard gas-induced pulmonary fibrosis [33]. Our results showed that serum levels of CXCL5, CXCL10, CCL2, CCL4, CCL5, and M-CSF were significantly increased in the SM group, while treatment with BMSCs significantly decreased these levels. CXCL5 is mainly induced by alveolar epithelial cells via Toll-like receptor 2 (TLR2). It induces chemotaxis and migration of neutrophils to the inflammatory site [34]. CXCL10 is believed to be a chemoattractant for T lymphocytes and neutrophils [35, 36]. CCL2 plays an important role in the initiation of inflammation [37]. The expression of CCR5 with its ligands CCL4 and CCL5 may act to promote the infiltration of T lymphocytes, monocytes, and neutrophils [38, 39]. Macrophage colony-stimulating factor, in addition to having key roles in the maturation, proliferation, and differentiation of macrophages, is also a potent inducer of macrophage migration [40]. It could be inferred that BMSC administration alleviates inflammatory cell infiltration partly via the inhibition of chemokines.

In addition to reducing the number of inflammatory cells, immune cell differentiation also plays an important role in immunomodulation. For example, there is a dichotomy in macrophage activation: M1 and M2, also known as classic and alternative, respectively. M1 macrophages are characterized by their ability to produce inflammatory responses, while M2 macrophages are characterized by their ability to antagonize inflammatory responses [41]. Additionally, CD4^+^ T lymphocytes, which consist of T helper (Th) 17 cells and regulatory T (Treg) cells, also play a key role in the pathogenesis or treatment of ALI [42, 43]. Th17 cells exert a powerful pro-inflammatory effect [44], while Treg cells have an anti-inflammatory effect due to the release of anti-inflammatory cytokines [45]. Previous research has evaluated the potential role of a Th17/Treg imbalance in the pathogenesis phase of sulfur mustard-induced lung injury [46]. Moreover, the role of macrophages in both the acute and long-term pulmonary injuries caused by sulfur mustard was also confirmed [47]. In our study, BMSC treatment significantly decreased the percentage of M1 macrophages and elevated the M2 macrophage level. Likewise, BMSCs could decrease the proportion of Th17 cells while increasing the proportion of Treg cells. These results suggest that the therapeutic role of BMSCs may be partly through their action on CD4^+^ T lymphocyte and macrophage subpopulation differentiation after SM exposure.

Previous studies have demonstrated that SM can upregulate various inflammatory cytokines [31]. In our study, reduced levels of the pro-inflammatory cytokines TNF-α, IL-1β, IL-6, IL-12, and IL-17 and significantly increased levels of the anti-inflammatory cytokines IL-10 and TGF-β were detected after BMSC treatment. Moreover, these results were also verified in cell coculture medium supernatant. Among these cytokines, TGF-β has an important effect on T cell differentiation. As an important cytokine in the development of Th17 and Treg cells, relatively low levels of IL-6 and high levels of TGF-β would mediate T cell differentiation toward the Treg subpopulation and away from the Th17 lineage [48]. As a primary effector molecule of Th17 cells, IL-17 appears to play a crucial role in activating and recruiting various immune cells during inflammation [49, 50], which could partly explain the serial decrease in Th17 cells, IL-17, and macrophages in our study.

The Toll-like receptor signaling pathway is reported to be essential for immunity; the activation of TLR4 leads to the activation of the NF-κB signaling pathway and the production of pro-inflammatory cytokines and chemokines by activating the innate immune system [51]. A previous study showed that activation of the Toll-like signaling pathway had an important role in inflammation and apoptosis after sulfur mustard exposure [52]. In our study, we found that exposure to sulfur mustard resulted in the elevated expression of the TLR4 protein and subsequent NF-κB signaling (p65/p50). Consistent with these results, the treatment with BMSCs and the transfection of macrophages with TLR4 siRNA or the TLR4 inhibitor TAK242 could significantly decrease TLR4 expression in macrophages and suppress the inflammation caused by sulfur mustard. TLR4 knockout mice were then used to confirm the mechanism, and we observed that the pro-inflammatory cytokine secretion induced by SM was significantly decreased when TLR4 was knocked out. These results demonstrated that BMSCs could suppress NF-κB activation through the regulation of the TLR4 signaling pathway in SM-induced lung injury.

In addition to exhibiting immunomodulatory activity, BMSCs were also capable of repairing lung injury by secreting different growth factors that can regulate epithelial permeability and enhance tissue repair [19]. Several growth factors were reported to have an important role in reducing alveolar edema [53–55]. In our study, EGF and PDGF levels after SM exposure were markedly reduced. All of these growth factors could be significantly increased after BMSC injection. EGF has been proven to increase cell proliferation and protect alveolar epithelial function [56, 57]. An impaired alveolar epithelial barrier was reported to increase mortality in an animal model of lung injury; however, FGF was beneficial for epithelial recovery due to its ability to maintain epithelial integrity and barrier function after lung injury [58]. PDGF was able to increase extracellular matrix deposition and promote pulmonary fibrosis formation [59]. These therapeutic effects could be revealed not only by decreased W/D ratio but also by Ki-67 protein staining and alveolar epithelial cell marker detection [60].

The alveolar epithelial cells are composed of two different cell types: type I alveolar epithelial cells (AEC I) and type II alveolar epithelial cells (AEC II). AEC I cells participate in the gas exchange between the blood and alveoli [61]. ACE II cells reduce surface tension and protect the alveoli from collapse [62]. Previous research reported that, due to increased capillary permeability, protein-rich edema fluid accumulates and results in the increasing movement of fluid from the capillaries to the pulmonary interstitial tissue [63]. As evidenced in our results, exposure to sulfur mustard decreased the expression of AQP-5 and SP-C, indicating the destruction of alveolar epithelial cells. Treatment with BMSCs significantly enhanced the cell proliferation of AECs, suggesting the role of BMSCs in promoting AEC cell recovery.

Regarding the increased W/D weight ratio and total protein, we speculate that SM poisoning may impair barrier function and eventually lead to perivascular edema. Decreased expression of VE-cadherin, Occludin, Claudin-5, and ZO-1 can significantly increase endothelial permeability, cause pulmonary edema, and aggravate lung injury [64, 65].VE-cadherin can further enhance tight junction formation by promoting the expression of Claudin-5 [66]. In our experiment, we found that compared with the SM group, BMSC treatment could significantly increase the expression of the above proteins, which fully demonstrated that BMSCs could reduce the severity of pulmonary edema by improving the integrity of the pulmonary barrier function.

## Conclusion

**Fig. 8:**
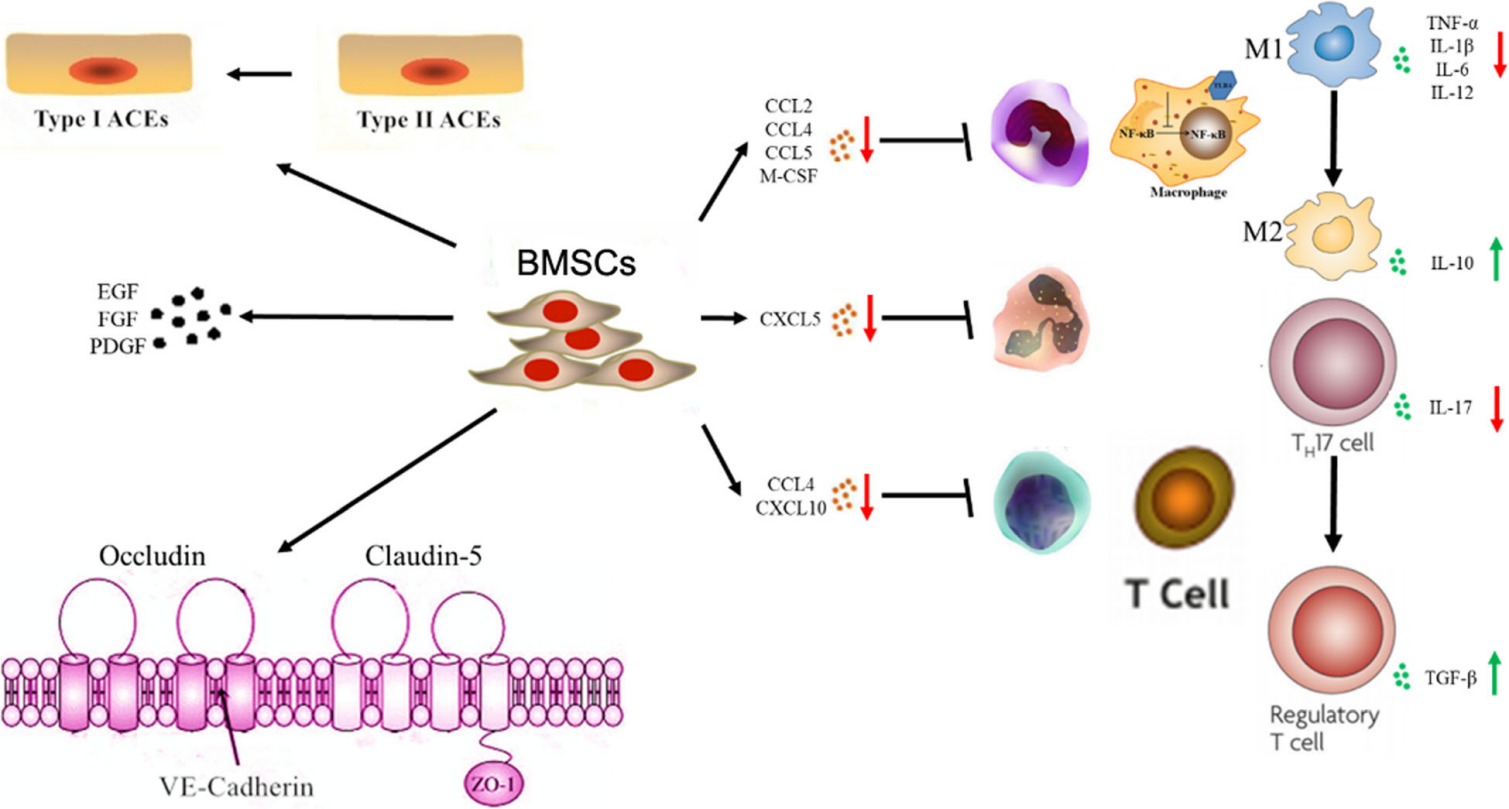
Model of the anti-inflammatory and tissue repair effects of BMSCs on SM-induced lung injury in mice

In conclusion, our study demonstrated the systemic therapeutic roles of BMSCs, including anti-inflammatory, immunomodulatory, and pro-reparative effects, in SM-induced lung injury in mice for the first time (Fig. 8). BMSC treatment represents a new therapeutic method for SM poisoning. However, further studies are still eagerly awaited to investigate the potential mechanism of the therapeutic effect of BMSCs on SM-induced lesions.

## Additional files

Additional file 1: **Table S1.** List of antibodies used in the study. (PDF 231 kb)

Additional file 2: **Figure S1.** Evaluation of respiratory function. (PDF 298 kb)

Additional file 3: **Figure S2.** (A) Analysis of the polarization of M1 macrophages by flow cytometry. *X*-axis: PE, anti-F4/80; *y*-axis: APC, anti-CD86. (B) Analysis of the polarization of M2 macrophages by flow cytometry. *X*-axis: PE, anti-F4/80; *y*-axis: APC, anti-CD206. (TIF 6165 kb)

Additional file 4: **Figure S3.** (A) Analysis of the polarization of Th17 cells by flow cytometry. *X*-axis: APC, anti-CD4; *y*-axis: PE, anti-RORγT (B) Analysis of the polarization of Treg cells by flow cytometry. *X*-axis: APC, anti-CD4; *y-*axis: PE, anti- FoxP3. (TIF 6473 kb)

## Abbreviations

AEC I: Type I alveolar epithelial cells
AEC II: Type II alveolar epithelial cells
ALI: Acute lung injury
BMSCs: Bone marrow-derived mesenchymal stromal cells
CCR2: CC chemokine receptor 2
CXCR2: CXC chemokine receptor 2
DMEM/F12: Dulbecco’s modified eagle medium/F12
EGF: Epidermal growth factor
ELISA: Enzyme-linked immunosorbent assay
FGF: Fibroblast growth factor
H&E: Hematoxylin-eosin
HRP: Horseradish peroxidase
MFI: Median fluorescent intensity
PBS: Phosphate buffer saline
PDGF: Platelet-derived growth factor
PEF: Peak expiratory flow
PIF: Peak inspiratory flow
PVDF: Polyvinylidene fluoride
SDS-PAGE: Sodium dodecyl sulfate-polyacrylamide gel electrophoresis
siTLR4: Small interfering RNA
SM: Sulfur mustard
SN-BMSCs: BMSCs supernatants
Th: T helper
TLR2: Toll-like receptor 2
TLR4: Toll-like receptor 4
Treg: Regulatory T
W/D ratio: Wet-to-dry lung weight ratio
