# Supplementary material for: The therapeutic effects of bone marrow-derived mesenchymal stromal cells in the acute lung injury induced by sulfur mustard
Source: Stem Cell Res Ther. 2019 Mar 12;10:90. doi: 10.1186/s13287-019-1189-x (PMC6416968; doi:10.1186/s13287-019-1189-x)
Supplement: Supplementary file 1 — Table S1. List of antibodies used in the study. (PDF 231 kb) [file 13287_2019_1189_MOESM1_ESM.pdf]

Table S1. List of antibodies

Name	Supplier	Catalog Number	Dilution ratio
CD11b	Biolegend	101207	1:80
CD19	Biolegend	152407	1:160
CD73	Biolegend	127205	1:80
CD90	Biolegend	105307	1:80
CD105	Biolegend	120413	1:80
CD34	Biolegend	119309	1:20
CD45	Biolegend	110713	1:80
CD3	Servicebio	GB11014	1:100
CD68	Servicebio	GB11067	1:100
Ki-67	Ebioscience	14-5698-80	1:300
F4/80	Biolegend	123110	1:20
CD86	Biolegend	105012	1:80
CD206	Biolegend	141707	1:40
CD4	Biolegend	100412	1:80
FoxP3	Biolegend	126404	1:20
RORγT	eBioscience	14-6981-80	1:80
Isotype Ctrl IgG 1	Biolegend	400407	1:80
Isotype Ctrl IgG 2a	Biolegend	400511	1:80
Isotype Ctrl IgG 2b	Biolegend	400607	1:80
TLR4	Proteintech	19811-1-AP	1:500

NF-κB p50	CST	#13586	1:1000
NF-κB p65	CST	#8242	1:1000
AQP-5	Abcam	ab78486	1:1000
SP-C	Abcam	ab211326	1:1000
VE-cadherin	Abcam	ab33168	1:500
Occludin	Abcam	ab167161	1:2000
Claudin-5	Abcam	ab15106	1:1000
ZO-1	Abcam	ab96587	1:1000
GAPDH	CST	#2118	1:1000
Goat anti-Mouse IgG (H+L) Poly-HRP Secondary Antibody, HRP conjugate	Pierce	32230	1:2000
Goat anti-Rabbit IgG (H+L) Poly-HRP Secondary Antibody, HRP conjugate	Pierce	32260	1:2000
