# Supplementary material for: The therapeutic effects of bone marrow-derived mesenchymal stromal cells in the acute lung injury induced by sulfur mustard
Source: Stem Cell Res Ther. 2019 Mar 12;10:90. doi: 10.1186/s13287-019-1189-x (PMC6416968; doi:10.1186/s13287-019-1189-x)
Supplement: Supplementary file 2 — Figure S1. Evaluation of respiratory function. (PDF 298 kb) [file 13287_2019_1189_MOESM2_ESM.pdf]

Evaluation of the respiratory function

Methods

The mice through different treatments were observed and compared carefully for 2 weeks. The change of respiratory function was detected by EMKA pulmonary system, the profile covers the following parameters peak expiratory flow, peak inspiratory flow, minute volume and 50% maximum expiratory flow velocity. The pulmonary function of mice was checked at different time points of 1d, 3d, 5d, 7d, 10d and 14d postinjection.

Results

The pulmonary function of mice was checked at different points of 1d, 3d, 5d, 7d, 10d and 14d post BMSCs injection. Compared with the control group, the peak expiratory flow, peak inspiratory flow, minute volume and 50% maximum expiratory flow velocity of SM-group mice was decreased. The injection of BMSCs produced significant increase on these indexes, which indicated BMSCs treatment would improve lung function in mice with SM poisoning (Figure S1).

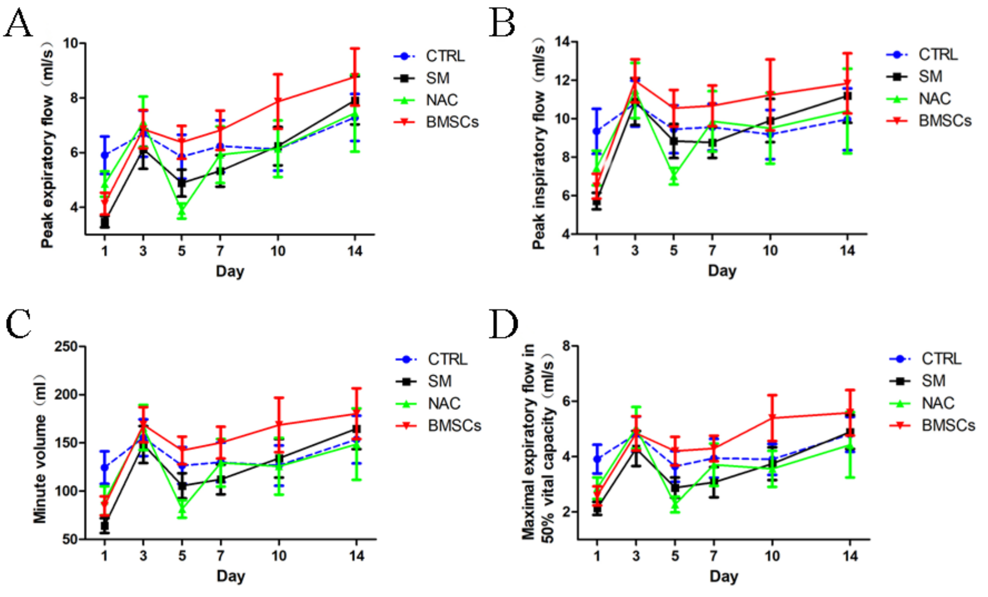

Figure S1. BMSCs protect against SM-induced mice on respiratory function evaluation. (A) Peak expiratory flow; (B) Peak inspiratory flow; (C) Minute volume; (D) 50% maximum expiratory flow velocity.
