# Supplementary figures and images for: The therapeutic effects of bone marrow-derived mesenchymal stromal cells in the acute lung injury induced by sulfur mustard
Source: Stem Cell Res Ther. 2019 Mar 12;10:90. doi: 10.1186/s13287-019-1189-x (PMC6416968; doi:10.1186/s13287-019-1189-x)

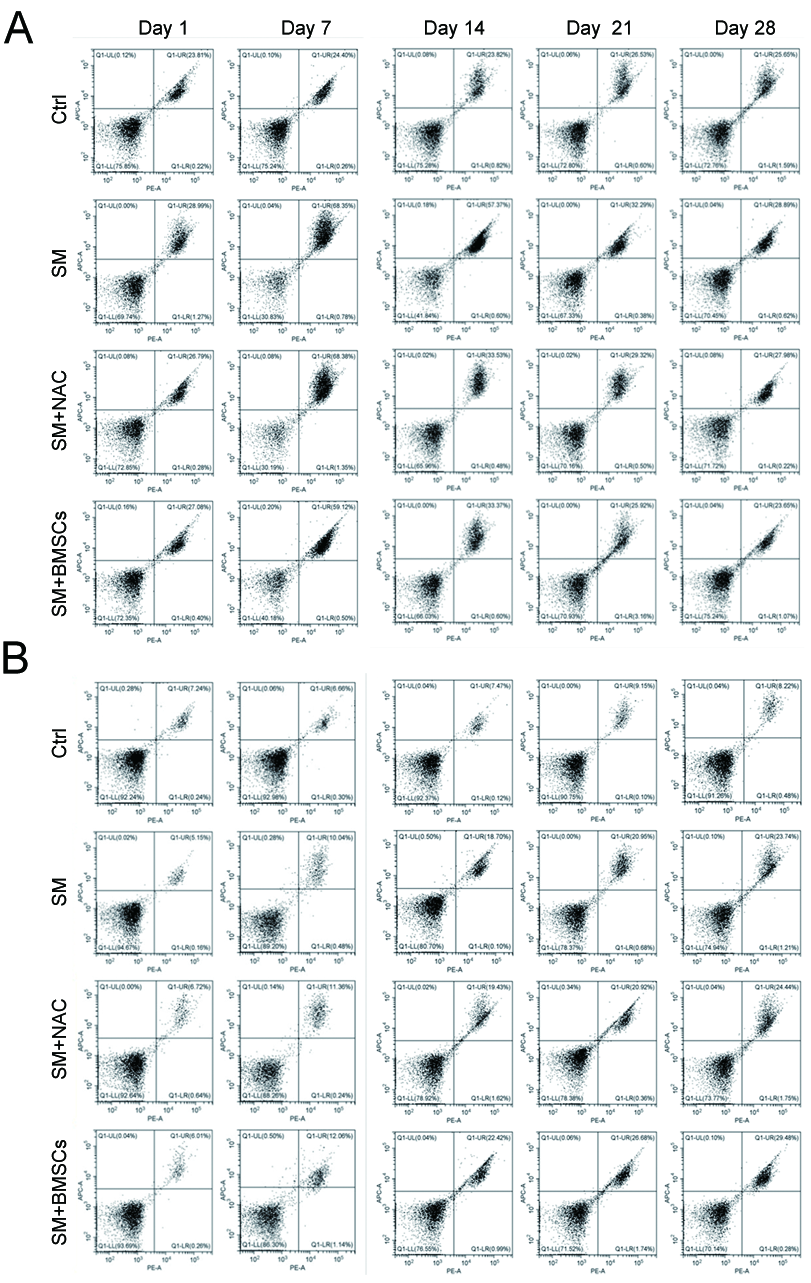

Supplement: Supplementary file 3 — Figure S2. (A) Analysis of the polarization of M1 macrophages by flow cytometry. X-axis: PE, anti-F4/80; y-axis: APC, anti-CD86. (B) Analysis of the polarization of M2 macrophages by flow cytometry. X-axis: PE, anti-F4/80; y-axis: APC, anti-CD206. (TIF 6165 kb) [file 13287_2019_1189_MOESM3_ESM.tif]

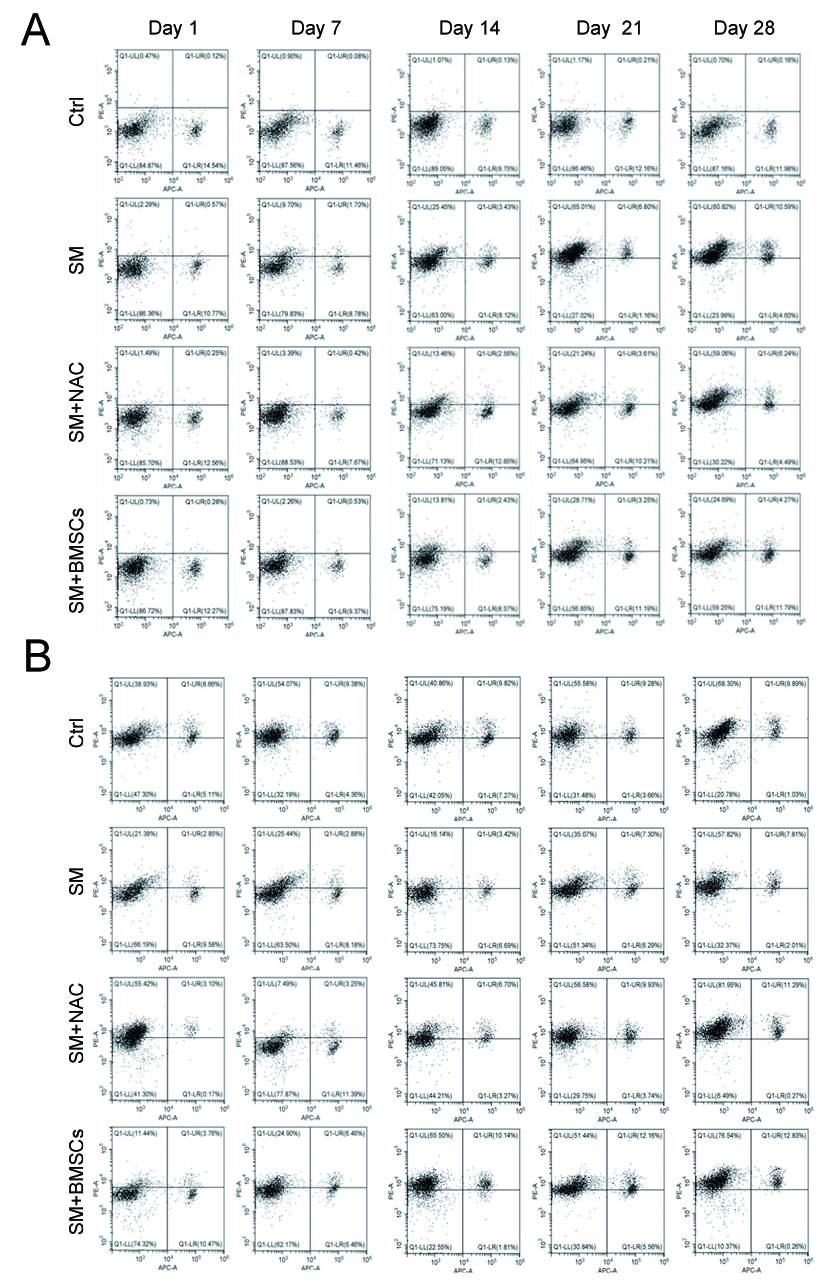

Supplement: Supplementary file 4 — Figure S3. (A) Analysis of the polarization of Th17 cells by flow cytometry. X-axis: APC, anti-CD4; y-axis: PE, anti-RORγT (B) Analysis of the polarization of Treg cells by flow cytometry. X-axis: APC, anti-CD4; y-axis: PE, anti- FoxP3. (TIF 6473 kb) [file 13287_2019_1189_MOESM4_ESM.tif]
